# Electric‐Field‐Driven Printed 3D Highly Ordered Microstructure with Cell Feature Size Promotes the Maturation of Engineered Cardiac Tissues

**DOI:** 10.1002/advs.202206264

**Published:** 2023-02-13

**Authors:** Guangming Zhang, Wenhai Li, Miao Yu, Hui Huang, Yaning Wang, Zhifeng Han, Kai Shi, Lingxuan Ma, Zhihao Yu, Xiaoyang Zhu, Zilong Peng, Yue Xu, Xiaoyun Li, Shijun Hu, Jiankang He, Dichen Li, Yongming Xi, Hongbo Lan, Lin Xu, Mingliang Tang, Miao Xiao

**Affiliations:** ^1^ Shandong Engineering Research Center for Additive Manufacturing Qingdao University of Technology Qingdao 266520 P. R. China; ^2^ Institute for Cardiovascular Science & Department of Cardiovascular Surgery of the First Affiliated Hospital Medical College Soochow University Suzhou 215000 P. R. China; ^3^ State Key Laboratory for Manufacturing System Engineering Xi'an Jiaotong University Xi'an 710049 P. R. China; ^4^ Department of Spinal Surgery The Affilliated Hosepital of Qingdao University Qingdao 266003 P. R. China; ^5^ Yantai Affiliated Hospital Binzhou Medical University Yantai 264100 P. R. China; ^6^ Institute of Rehabilitation Engineering Binzhou Medical University Yantai 264100 P. R. China; ^7^ Co‐innovation Center of Neuroregeneration Nantong University Nantong 226001 P. R. China

**Keywords:** 3D highly ordered microstructure, cell feature size, electric‐field‐driven, engineered cardiac tissues, small fiber spacing

## Abstract

Engineered cardiac tissues (ECTs) derived from human induced pluripotent stem cells (hiPSCs) are viable alternatives for cardiac repair, patient‐specific disease modeling, and drug discovery. However, the immature state of ECTs limits their clinical utility. The microenvironment fabricated using 3D scaffolds can affect cell fate, and is crucial for the maturation of ECTs. Herein, the authors demonstrate an electric‐field‐driven (EFD) printed 3D highly ordered microstructure with cell feature size to promote the maturation of ECTs. The simulation and experimental results demonstrate that the EFD jet microscale 3D printing overcomes the jet repulsion without any prior requirements for both conductive and insulating substrates. Furthermore, the 3D highly ordered microstructures with a fiber diameter of 10–20 µm and spacing of 60–80 µm have been fabricated by maintaining a vertical jet, achieving the largest ratio of fiber diameter/spacing of 0.29. The hiPSCs‐derived cardiomyocytes formed ordered ECTs with their sarcomere growth along the fiber and developed synchronous functional ECTs inside the 3D‐printed scaffold with matured calcium handling compared to the 2D coverslip. Therefore, the EFD jet 3D microscale printing process facilitates the fabrication of scaffolds providing a suitable microenvironment to promote the maturation of ECTs, thereby showing great potential for cardiac tissue engineering.

## Graphical abstract

A simple, and efficient strategy using electric‐field‐driven jet microscale 3D printing to fabricate 3D highly ordered microstructures with both the fiber width and fiber spacing that match myocardial feature sizes is first developed to build engineered cardiac tissues (ECTs) with hiPSC‐CMs. The myocardial feature‐sized structure promoted the maturation of ECTs, thereby showing great potential for cardiac tissue engineering.

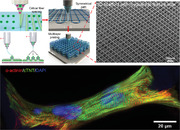

## 1 Introduction

Cardiovascular disease continues to be a major cause of morbidity and mortality worldwide.^[^ ^1^ ^]^ However, donor organs are in short supply and the adult myocardium has a limited regenerative capacity. Cell therapy and cardiac tissue engineering are viable solutions to support the regeneration of cardiac tissue. These solutions offer an improved quality of life for patients with cardiovascular diseases.^[^ ^2^, ^3^, ^4^, ^5^ ^]^ Human‐induced pluripotent stem cell‐derived cardiomyocytes (hiPSC‐CMs) are great options for building engineered cardiac tissues (ECTs). This allows us to replace dysfunctional cardiomyocytes and build cardiovascular disease models for drug screening and also cardiotoxicity testing.^[^ ^6^, ^7^, ^8^ ^]^ However, the predictive powers of these models are limited by the immature state of the ECTs. Many studies have been conducted to improve the maturity of hiPSC‐CMs and ECTs to overcome this limitation.^[^ ^9^ ^]^ Besides the biochemical stimulations, physical cues are also crucial.^[^ ^10^ ^]^ Scaffolds are the base of cardiac tissue engineering, which must provide an appropriate microenvironment for the maturation and function of hiPSC‐CMs. A variety of synthetic and natural biomaterials have been developed to fabricate scaffolds that can mimic the cellular microenvironment for the growth and maturation of hiPSC‐CMs.^[^ ^11^, ^12^, ^13^ ^]^ And the architectures of scaffolds, such as fiber diameter, fiber spacing, degree of porosity, and fiber alignment have been found to influence the cell fate^[^ ^14^, ^15^, ^16^ ^]^ and are crucial for ECT maturation. Nicole et. al fabricated micro‐scaffolds with myocardium's feature morphology so that the pattern promoted the maturation of single hiPSC‐CM.^[^ ^17^ ^]^ Furthermore, 3D porous structures have been proven to facilitate the maturation of 3D ECTs.^[^ ^18^ ^]^ Therefore, to integrate the 3D property of tissues and also the myocardium's feature morphology, using scaffolds with a 3D highly ordered microstructure and feature size (both fiber diameter and fiber spacing) similar to the native myocardium may be a potential method to promote ECT maturation.

Electrohydrodynamic (EHD)‐based jet printing methods, such as electrospinning and melt electrowriting (MEW) has been considered a powerful choice to create highly complex and multicomponent structures with well‐defined architectures.^[^ ^19^, ^20^, ^21^, ^22^ ^]^ Currently, lots of reported scaffolds prepared by EHD‐based jet printing have been successfully applied for in vivo and in vitro experiments.^[^ ^23^, ^24^, ^25^, ^26^ ^]^ Although the fiber diameter (10–20 µm) has been printed sufficiently suitable for cell attachment,^[^ ^27^, ^28^ ^]^ most of the developed scaffolds usually possess fiber spacing of more than 200 µm, which is much larger than the width of myofibers within the myocardial tissue (about 30 to 40 µm).^[^ ^18^ ^]^ It is still challenging to fabricate 3D highly ordered microstructures with both suitable fiber diameter and fiber spacing due to the inherent mechanism of conventional EHD‐based jet printing.^[^ ^29^, ^30^ ^]^ The major challenge for the precise deposition of the jet is mainly ascribed to the jet repulsion by the residual charges from field‐induced emission and charge injection mechanism.^[^ ^31^, ^32^ ^]^ Residual charges in conductive printing materials can be transferred to the conductive substrate, or eliminated by neutralization using an AC power supply on the insulating substrate.^[^ ^33^ ^]^ However, the semi‐conductive nature of melt biodegradable polymers results in residual charges being entrapped in the scaffold. This complicates the order of the microstructure and prevents precise deposition.^[^ ^31^ ^]^ To solve this problem, efforts have been made to minimize the achievable fiber spacing for a fixed fiber diameter by adjusting the conductivity of the substrate/printing materials,^[^ ^31^, ^33^ ^]^ and forcing the jet direction using an auxiliary electrode.^[^ ^34^ ^]^ These methods are effective while being inflexible and cannot solve the residual charge problems from the source. When the printing conditions (printing materials, printing substrates, and designed structure) are changed, the influence behavior of residual charges will also change, thus increasing the difficulty of regulation. In addition, even if the jet repulsion caused by the residual charge is eliminated, the jet will be attracted and stacked on the printed structure because of the polarization of the molten polymer exposed in the electric field.^[^ ^35^ ^]^ Therefore, how to conquer the jet deflection to achieve 3D highly ordered microstructures with cell feature sizes remains a major challenge.

In this work, we used an electric‐field‐driven (EFD) printed 3D highly ordered microstructure with cell feature size (fiber diameter of 10–20 µm and fiber spacing of 60–80 µm) for promoting the maturation of ECTs. Benefiting from the cancellation of the ground electrode, EFD jet microscale 3D printing not only possesses better electric field stability for multilayer 3D printing,^[^ ^36^ ^]^ but also overcomes the jet repulsion effect for both conductive and insulating substrate, which lays a good foundation for precise deposition. Therefore, 3D highly ordered microstructures with small fiber spacing can be fabricated by maintaining a vertical jet by overcoming jet deflection and using a symmetrical printing path. Finally, we investigated the potential applications of these microstructures in cardiac tissue engineering. The results indicated the potential applicability of 3D‐printed scaffolds in the biomedical field and their ability to promote cardiomyocyte maturation.

## 2 Results and Discussion

**Figure 1:**
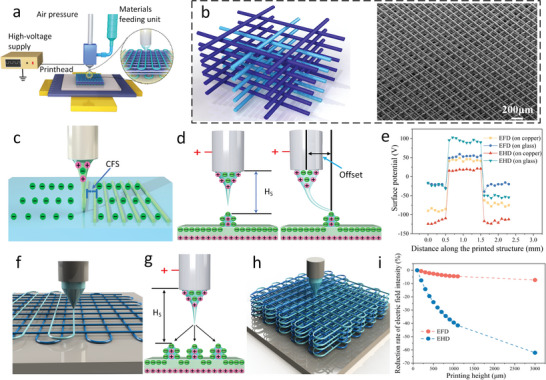
a) The set‐up schematic of EFD jet 3D printing. b) Designed and printed 3D highly ordered microstructure with a cell feature size (line width of 10–20 µm and fiber spacing of 60–80 µm). c) Critical fiber spacing (CFS) for precise printing of single‐layer grid structure in the case of no jet deflection. d) Only the charge‐induced self‐alignment happens in EFD jet microscale 3D printing where the jet can be attracted by the printed structure. e) The surface potential of deposited structures printed by EFD jet 3D printing and EHD jet printing on both copper and glass substrates. f) Reducing fiber spacing of the printed grid by using a symmetrical printing path, and the jet deflection will be restrained in the condition that printing in the middle of a pair of symmetrical walls. g) The charges distribution of the substrate with symmetrical printed structures. h) Printing highly ordered multilayer microstructures. i) Reduction rate of electric field intensity in EHD‐based jet printing and EFD jet microscale 3D printing.

The fabrication procedure for the 3D highly ordered microstructure of polylactic acid (PLA) with cell feature size by EFD jet microscale 3D printing is explained schematically (**Figure** **1**). Differing from traditional EHD jet printing with two electrodes, the ground electrode (a conducting substrate or mounted under an insulating substrate) has been canceled in EFD jet microscale 3D printing system (Figure 1a). Therefore, the generation method of the driven electric field in the EFD jet microscale printing is mainly based on the charge polarization between the nozzle tip and the top surface of the substrate/printed structure^[^ ^37^ ^]^ (Figure S1, Supporting Information). To achieve the cell feature size for ECTs, the 3D highly ordered microstructure is defined as a fiber diameter of 10–20 µm and fiber spacing of 60–80 µm (Figure 1b), according to the width of the myofibers within the myocardial tissue (≈30 to 40 µm).^[^ ^18^ ^]^ The fabrication procedure is then divided into three steps: a) The first step is to print a single‐layer grid structure with critical fiber spacing (CFS) in the case of precise deposition (Figure 1c). To achieve this goal, on one hand, the jet repulsion should not occur, which inevitably happens in EHD‐based printing due to the residual charges trapped in the printed structure, resulting in a very large CFS of more than 200 µm. However, EFD jet microscale 3D printing overcomes the jet repulsion and makes the jet attraction happen on both conductive and insulating substrates, which is defined as the charge‐induced self‐alignment effect (CISA) (Figure 1d). This mainly attributes to the much fewer residual charges transferred from the nozzle electrode to the printed structure and a stronger polarization effect, which can be proved by the evidence that the printed structure of PLA on both copper and glass substrates presents a very similar surface potential, which is larger and lower than the surface potential of EHD‐based printed PLA structure on the copper and glass substrate, respectively (Figure 1e). On the other hand, the CISA effect should also be overcome, and a CFS should be determined to ensure that the jet is vertical to the substrate to maintain uniform fiber spacing. Otherwise, the deflected jet will seriously affect the microstructure order. b) The second step is to further reduce the fiber spacing of the printed grid (Figure 1f). In this step, a symmetrical path scheme was used for printing in the middle of two previously printed adjacent fibers, and the effect of electrostatic forces on the jet was balanced by the two symmetrical printed structures, thereby allowing the jet to be oriented vertically downward to the substrate and deposit precisely according to the predetermined path (Figure 1g). c) The third step is to print 3D multilayer microstructures (Figure 1h). With the height of the printed structure increasing from 0 to 3000 µm, the electric field in EFD jet microscale 3D printing just decreases by less than 10%, while that of EHD jet printing decreases by more than 60% (Figure 1i and Figure S2, Supporting Information). The higher electric field stability of EFD jet microscale 3D printing ensures the stable jet for multilayer 3D printing (Figure S3 and Table S1, Supporting Information).^[^ ^36^ ^]^ Therefore, a 3D highly ordered microstructure with a cell feature size can be achieved.

### 2.1 Principle of Charge‐Induced Self‐Alignment

The jet flow rate, which multiplies the ejection volume of printing materials with the jet duration, is well known to play a major role in EHD‐based jet printing,^[^ ^38^ ^]^ which can be written as,

(1) $$Q=\frac{\pi D_{n}^{4}}{128\mu L_{n}}\left(\Delta P+\frac{1}{2}\varepsilon_{0}E^{2}-\frac{2\gamma }{D_{n}}\right)$$

where *µ* is the viscosity of the printing material, *D_n_* and *L_n_* are the inner diameter and length of the nozzle, respectively, *ΔP* is the air pressure, *ε_0_* is the permittivity of vacuum, *E* is the magnitude of the external electric field, and *γ* is the surface tension of the air‐liquid interface. During the printing process, the air pressure (*ΔP*) and electric force (*ε*_0_ *E* ^2^/2) overcome the capillary pressure (2*γ*/*D_n_*) to drive the flow through the small nozzle. The air pressure (*ΔP*) is usually used to ensure proper material supply by overcoming the capillary pressure (surface tension of the air‐liquid interface) to form a meniscus on the tip of the nozzle, where the air pressure is equal to the capillary pressure in the equilibrium state. Therefore, the shape and direction of the cone jet mainly depend on the value and direction of the electric field force, respectively.

**Figure 2:**
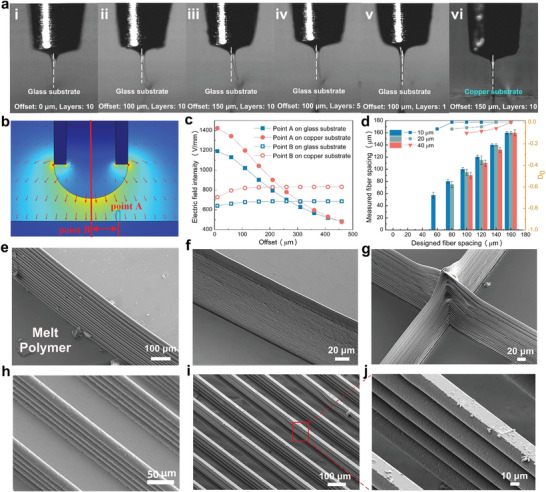
a) Jet behaviors observed by the charge‐coupled device (CCD) camera in the cases of jet on the 10 stacked layers with offsets of i) 0, ii) 100, and iii) 150 µm; jet with an offset of 100 µm on ii)10 stacked layers, iv) 5 stacked layers, and v) 1 stacked layer; jet on 10 stacked layers with an offset of 150 µm on iii) glass, and vi) copper substrates; b) Simulation results of the electric field distribution between a nozzle and a printed structure (20 µm width and 60 µm height) for different offsets on copper and glass substrates. c) Simulation results of the electric field intensities at the top center of the printed structure (Point A) and the substrate (Point B) (copper and glass substrates). d) Fiber deposition results on the copper and glass substrates. Scanning electron microscope (SEM) morphologies of the thin‐walled structures with increasing ARs: e) 6.78, f) 21.8, g) 28.2. SEM morphologies of the printed multilayer grid structure: h) fiber diameter of 10 µm and fiber spacing of 80 µm. i,j) fiber diameter of 20 µm and fiber spacing of 140 µm.

To quantitatively analyze the CISA effect, different offsets (the horizontal distance from the nozzle to the printed structure), layers of the printed structure, and substrate were investigated (**Figure** **2**a). When the offset increases from 0 to 150 µm, the jet tends to deposit on the top surface of 10 stacked layers with an increasing deflection angle (Figure 2a(i)–(iii) and Video S1, Supporting Information), whereas the jet is deposited on the substrate when the offset is larger than 200 µm (Figure S4, Supporting Information). The jet is almost perpendicular to the substrate with the 1‐layer structure and deflected towards the 5‐layer structure with the heights of the printed structures in the range of 0–60 µm (1, 5, and 10 layers) at an offset of 100 µm (Figure 2a(ii),(iv),(v)). This demonstrates that the jet deflection is closely related to the distance between the nozzle and the printed structure. When the offset is small or the printed structure is high, the electric field force generated between the nozzle and the top surface of the printed layer is much greater than that generated between the substrate and nozzle, and then the jet deflects to the top surface of the printed structure. A multi‐jet can occur (Figure S5, Supporting Information) even under specific conditions with two printed structures. In addition, the jet is deposited on the conductive copper substrate while deflecting to the top surface of the 10‐layer structure on insulating glass under the same offset of 150 µm (Figure 2a–(v),(vi)). Therefore, the material properties of the substrate also influence the CISA stacking owing to the charge polarization/induction phenomena and residual charge transfer. The jet reflection depends on the trade‐off between the electric field intensity of the printed structure and the substrate, which is influenced by the height, offset, and material properties. We then simulated the variation trend of the electric field intensity of adjacent printed PLA structures (width of 20 and height of 60 µm) with different offsets on both copper and glass substrates (Figure 2b). The electric field intensity of the top center of the printed structure (point A) and the substrate (point B) at different offsets are shown (Figure 2c). The electric field intensity of Point A showed a negative correlation change with the increase in offset, whereas that of point B showed a slightly increasing trend. The electric field intensities of points A and B can be equal at an offset in the range of 200–300 µm for both substrates, which is consistent with the experimental results. Furthermore, the degree of offset (*Do*) of the fiber with a diameter in the range of 10–40 µm deposited on the glass substrate is shown in Figure 2d to describe the migration of fibers during deposition and determine the CFS for the vertical jet. *Do* passes through three stages for all three fiber diameters: multilayer stacking by CISA, jet deflection, and precise deposition (Figure S6, Supporting Information). For example, when the designed fiber spacing increased from 0–160 µm, a fiber with a diameter of 10 µm stacked on the previous fiber when the designed fiber spacing was smaller than 60 µm. Furthermore, a negative *Do* was obtained with fiber spacing in the range of 60–100 µm, thereby achieving precise deposition according to the designed value when the fiber spacing was larger than 100 µm (Figure S7, Supporting Information). This also confirmed that only an attraction effect existed between the jet and printed structure on the insulating substrate in EFD microscale 3D printing. Therefore, two typical cases were demonstrated: 1) a multilayer stacked melt polymer of PLA on an insulating substrate owing to the CISA effect. Figure 2e–g shows three thin‐walled structures made of PLA with different ARs on a glass substrate. The AR increases from 6.78 in Figure 2e to 21.8 in Figure 2f and finally, to 28.2 in Figure 2g with precise stacking from several layers to more than 40 layers. 2) The grid structures overcame the CISA effect. Figure 2h–j shows the scanning electron microscope (SEM) morphologies of the printed multilayer grid structure: fiber diameters of 10 and 20 µm and fiber spacings of 80 and 140 µm (Figure 2h,i).

### 2.2 Printing 3D Highly Ordered Microstructure with Small Fiber Spacing

**Figure 3:**
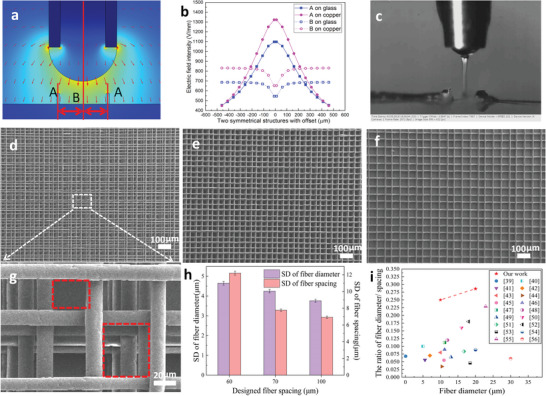
Main factors for printing 3D highly ordered microstructures: a) simulation results of the electric field distribution, b) simulation results of the electric field intensity with a pair of symmetrical structures, and c) the jet deflection is restrained when printing in the middle of a pair of symmetrical structures. d) SEM morphologies of 3D highly ordered microstructures with a fiber diameter of 20 µm and fiber spacings of d,g) 60, e) 70, and f) 100 µm. h) Standard deviation of the fiber diameter and fiber spacing versus designed fiber spacing. i) Comparison between the fiber diameter/fiber spacing obtained in this study with other state‐of‐the‐art results reported in the literature (Zou et al.,^[^ ^39^ ^]^ Kong et al.,^[^ ^40^ ^]^ Wang et al.,^[^ ^41^ ^]^ Bertlein et al.,^[^ ^42^ ^]^ Juliane et al.,^[^ ^43^ ^]^ Eichholz et al.,^[^ ^44^ ^]^ Li et al.,^[^ ^45^ ^]^ Hrynevich et al.,^[^ ^46^ ^]^ Zhang et al.,^[^ ^47^ ^]^ Chen et al.,^[^ ^48^ ^]^ Delalat et al.,^[^ ^49^ ^]^ He et al.,^[^ ^50^ ^]^ Gwiazda et al.,^[^ ^51^ ^]^ Lourdes et al.,^[^ ^52^ ^]^ Bas et al.,^[^ ^53^ ^]^ Fuchs et al.,^[^ ^54^ ^]^ Xie et al.,^[^ ^55^ ^]^ Hochleitner et al.^[^ ^56^ ^]^)

The strong CISA stacking effect in EFD jet 3D printing has been proven to benefit the fabrication of high‐AR microstructures on both conductive and insulating substrates. The CFS of the vertical jet was determined to achieve a precise deposition. However, the achieved CFS is still much larger than the cell feature size. Therefore, we proposed a symmetrical printing path to further reduce the fiber spacing by printing another fiber in the middle of two adjacent fibers. Considering that the electric field is the essential reason for the deflection of the jet, we simulated the change of the electric field in the presence of two printed structures and found that the direction of the electric field lines in the middle is almost perpendicular to the substrate (**Figure** **3**a). The electric field intensities were equal at the two locations (Figure 3b). Subsequently, we investigated the jet behavior under different offset parameters. The jet deflection was restrained when printing in the middle of a pair of symmetrical walls (Figure 3c and Video S2, Supporting Information), and the jet always deposited vertically on the substrate under the effect of the symmetrical electric field. Therefore, a highly ordered structure with small fiber spacing can be printed by maintaining the jet vertically downward to the substrate according to the two symmetrically distributed electric field forces. Subsequently, 3D highly ordered microstructures with different fiber spacings were demonstrated, including 10‐layer microstructures with a fiber diameter of 20 µm and spacing of 60 µm (Figure 3d), a fiber diameter of 20 µm and spacing of 70 µm (Figure 3e), and fiber diameter of 20 µm and spacing of 100 µm (Figure 3f). However, due to the CFS for precise deposition of the fiber with a diameter of 20 µm is 140 µm, the measured fiber spacing of microstructure deviates from the designed value of 60 µm with a large spacing of 70 µm and a small spacing of 50 µm (shown in Figure 3g). The SD of the measured fiber diameter and spacing was then calculated (Figure 3h). The SD of 12.2 for the designed fiber spacing of 60 µm is greater than 7.7 for a fiber spacing of 70 µm and 6.9 for a fiber spacing of 100 µm. Finally, we introduced and compared the ratio of fiber diameter/fiber spacing with other state‐of‐the‐art results in the literature (Figure 3i) to demonstrate the ability to fabricate 3D highly ordered microstructures with a very small fiber spacing. Our method can achieve the largest ratios of fiber diameter/fiber spacing, that is, 0.29 (20 µm) and 0.25 (10 µm), which are much larger than the reported values^[^ ^39^, ^40^, ^41^, ^42^, ^43^, ^44^, ^45^, ^46^, ^47^, ^48^, ^49^, ^50^, ^51^, ^52^, ^53^, ^54^, ^55^, ^56^ ^]^ (Table S2, Supporting Information). For example, He et al. fabricated a scaffold with a fiber diameter/fiber spacing of 0.16 using melt EHD printing,^[^ ^50^ ^]^ and Lourdes et al. achieved a ratio of 0.18 using MEW.^[^ ^52^ ^]^ Therefore, such highly ordered microstructures with such a large fiber diameter/fiber spacing ratio have not been reported before.

### 2.3 3D Printed Microstructure with Cell Feature Size Promoted the Maturation of ECTs

**Figure 4:**
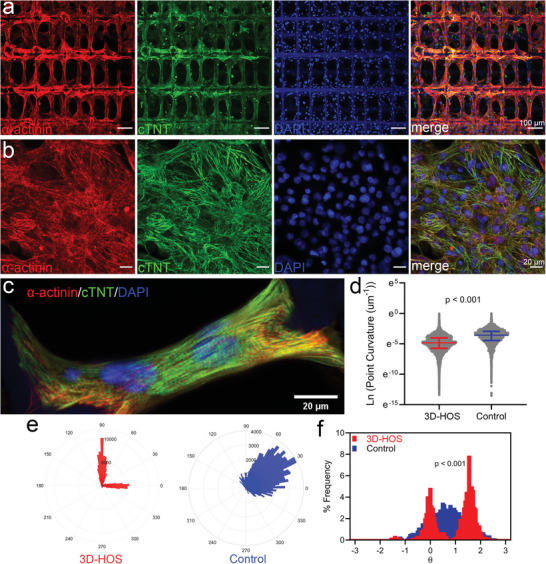
3D‐printed scaffolds directed the hiPSC‐CMs to align to develop oriented ECTs. The hiPSC‐CMs were seeded into 3D‐printed scaffolds and on glass coverslips after purification. After 10 days of culture in vitro, the hiPSC‐CMs were stained with alpha sarcomeric actinin (*α*‐actinin), and cardiac troponin T (cTNT). 4',6‐diamidino‐2‐phenylindole (DAPI) was used to stain the nucleus in a) 3D‐printed scaffolds and b) coverslip, respectively. c) 3D‐printed scaffolds had cardiomyocyte feature‐sized skeletons, which facilitated the orientation of hiPSC‐CMs and the morphology of ECTs with high magnification. d) Comparison of sarcomeric curvature values of hiPSC‐CMs cultured in 3D‐printed scaffolds and coverslip. Data were acquired from 19 442 sarcomeric points in 3D‐printed scaffolds and 36 852 sarcomeric points on the coverslip. The data was shown with a median (interquartile range). *p* < 0.001 from Mann–Whitney test. e) Representative polar histogram of the sarcomeric orientation of the hiPSC‐CMs cultured in 3D‐printed scaffolds and coverslips. f) Comparison of sarcomeric orientation distribution of hiPSC‐CMs cultured in the 3D‐printed scaffolds and coverslips. *p* < 0.001 from Kolmogorov–Smirnov test.

The native cardiac tissues comprise layers of aligned myocardium, which enables efficient excitation‐contraction coupling for synchronic contraction and efficient blood pumping. Therefore, the controllable alignment of hiPSC‐CMs in scaffolds may be a critical solution for the maturation of ECTs in cardiac tissue engineering.^[^ ^57^ ^]^ Additionally, hiPSC‐CMs were cultured in the scaffolds with the above‐mentioned 3D highly ordered microstructure (fiber diameter of 20 µm and fiber spacing of 80 µm) to develop oriented ECTs and demonstrate whether the abovementioned 3D highly ordered microstructure with small fiber spacing is suitable for potential cardiac tissue engineering applications. As previously described, hiPSC‐CMs are directed by temporal modulation of the canonical Wnt pathway.^[^ ^58^ ^]^ Cardiomyocytes were purified and seeded into 3D‐printed scaffolds on day 20 after differentiation with cells planted on coverslips as controls. Cardiomyocytes can easily and steadily attach to PLA fibers. Cardiomyocytes maintained good cellular viability and continuous autonomous contractility in both groups during cultivation for ten consecutive days. Immunostaining the 3D‐printed scaffolds with sarcomeric‐actinin and cardiac troponin T (cTNT) facilitated the orientation of hiPSC‐CMs, as shown in **Figure** **4**a,b. Cardiomyocytes were highly arranged and aligned with the microstructure along the PLA fibers. However, the sarcomeric orientation was completely random in the coverslip controls. Additionally, the 3D‐printed scaffolds with paved cardiomyocytes are approximately rectangular with a high aspect ratio, and the myofilament structure is organized (Figure 4c), thereby displaying a maturation trend. Furthermore, we calculated sarcomeric curvature values and analyzed sarcomeric orientation based on immunostaining. The curvature of a specific intermediate point corresponded to a tortuous structure. When sarcomeres have a tortuous morphology, their curvature is large.^[^ ^59^ ^]^ The small curvature value (Figure 4d) and highly centralized orientation (Figure 4e,f) in the 3D‐printed scaffold group revealed a relatively highly ordered structure. One typical feature of mature cardiomyocytes is their elongated, nearly cuboid shape and clearly distinguishable sarcomere,^[^ ^60^ ^]^ as shown in Figure 4c. The 3D‐printed scaffolds promoted the striation pattern of the ECTs with one long axis along which myofibrils are regularly arranged, thereby indicating the maturation of hiPSC‐CMs and their potential application in cardiac tissue engineering.

**Figure 5:**
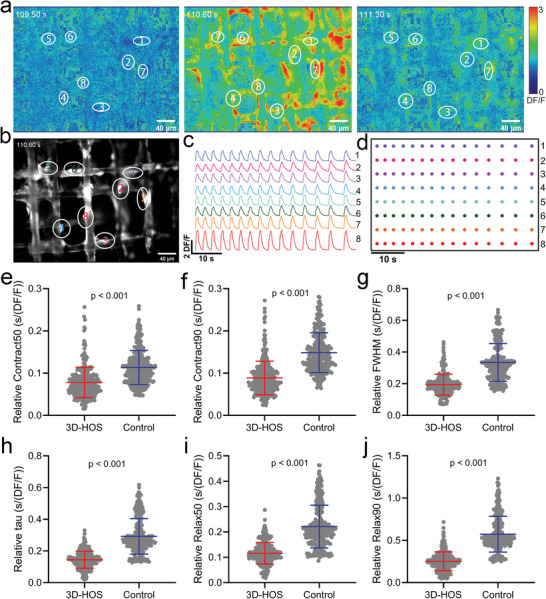
3D‐printed scaffolds directed the hiPSC‐CMs alignment to develop synchronous functional ECTs and promote their maturation. a) Representative DF/F fluorescent images in a time series of the ECTs formed in 3D‐printed scaffolds loaded with calcium fluorescent dye Fluo‐4 AM after 8 days of culture in vitro. The labeled numbers indicate the cardiomyocytes attached to the skeleton of the 3D‐printed scaffolds. b) Immunofluorescence images of cardiomyocytes loaded with calcium fluorescent dye Fluo‐4 AM. The labeled numbers were corresponding to (a). c) Synchronous calcium transients acquired from the labeled cardiomyocytes in (b). d) Raster plot indicates the peaks of the calcium transients acquired from labeled cardiomyocytes in (b). e–j) The calcium transient parameters including relative Contract50, Contract90, FWHM, tau, Relax50, and Relax90 were compared. Data were acquired from 297 transits in 3D‐printed scaffolds and 284 transits on the coverslip. The data was shown with a median (interquartile range). *p* < 0.001 from Mann–Whitney test.

Calcium plays a central role in cardiomyocyte function because it regulates the process of excitation‐contraction coupling, which links the electrical excitation of the plasma membrane to contraction. During hiPSC‐CM maturation, cardiac tissues develop highly efficient calcium handling to form a synchronous contraction.^[^ ^61^ ^]^ Furthermore, we monitored spontaneous calcium activity by fluorescence calcium imaging (Video S3, Supporting Information) to evaluate the calcium handling efficiency of our ECTs cultured in the 3D‐printed scaffolds. Fluo‐4 AM fluorescent images showed clear bright spots associated with hiPSC‐CMs (**Figure** **5**b). Additionally, the hiPSC‐CMs formed well‐defined connections along the PLA fibers. Subsequently, we conducted a time‐lapse analysis of intercellular calcium signaling. We also converted the Fluo‐4 AM fluorescence images to the color saturation mode to better show calcium transients (Figure 5a and Video S4, Supporting Information). As recorded the eight randomly picked regions attached to the scaffold in Figure 5a,b, shows the fluorescent calcium transient in synchrony over time as displayed by the change and distribution of fluorescence density from the whole. More specifically, calcium transients in the eight randomly selected regions exhibited orchestrated frequency and similar amplitude (Figure 5c,d), thereby indicating the development of synchronous and functional ECTs. The high efficiency of calcium handling serves as a solid indicator of the maturation of ECTs.^[^ ^57^ ^]^ Immature hiPSC‐CMs show slower calcium dynamics with an increased time to peak and slower decay of the calcium transient.^[^ ^61^ ^]^ The ECTs also showed functional synchronization when hiPSC‐CMs were cultured in the scaffolds for as long as 14 days (Figure S9 and Video S5, Supporting Information).

To verify if the 3D‐printed scaffolds could increase the efficiency of calcium handling of ECTs, the parameters of the full‐width half max (FWHM), constant Tau during decay, a half time during the rise (Contract50), 90% time during the rise (Contract90), a half time during the decay (Relax50) and 90% time during the decay (Relax90) (showing in Figure S11, Supporting Information) were calculated from the code provided by Prof. Gordana Vunjak‐Novakovic at Columbia University with some modifications.^[^ ^62^ ^]^ Since the ECTs developed in 3D‐printed scaffolds and 2D coverslips presented different amplitudes of the calcium transients, the parameters were normalized to the amplitude. Comparisons of the relative Contract50, Contract90, FWHM, Tau, Relax50, and Relax90 are shown in Figure 5e–j. Notably, all the parameters of the 3D‐printed scaffolds were significantly lower than those of the 2D coverslip, thereby indicating a higher efficiency of calcium handling of ECTs developed in 3D‐printed scaffolds than those of the 2D coverslip. Therefore, 3D‐printed scaffolds with such fiber diameters and fiber spacing promoted the maturation of ECTs.

Since myofibers are oriented in parallel in cardiac tissue, future work on tuning the length‐width ratios and angles between the layers will be carried out to allow more cells to grow in one direction. Scaffolds with length‐width ratios of 1:1, 1:1.5, and 1:2 have been fabricated, and hiPSC‐CMs were cultured in the scaffolds (Figure S8, Supporting Information). The maturation of ECTs will be investigated in future studies. Moreover, we tuned the angles (90°–45°) between the PLA layers to study their effects on the maturation of ECTs (Figure S8, Supporting Information). The angles between the PLA layers could affect the arrangement of hiPSC‐CMs. The cells oriented in parallel better at 45° than that at 90° (Figure S8d,e, Supporting Information). Furthermore, the ECTs developed in 3D PLA scaffolds can be applied in myocardial infarction (MI) treatment, patient‐specific disease modeling, and drug evaluations. Additionally, the 3D‐printed PLA scaffolds have been proven safe when used as cardiac patches (Figure S10, Supporting Information). In the meanwhile, caffeine, verapamil, and nifedipine are being tested in these ECTs.

In summary, we demonstrated a simple, and efficient strategy using EFD jet microscale 3D printing to fabricate 3D highly ordered microstructures with both the fiber width and fiber spacing that match myocardial feature sizes to promote the maturation of ECTs. The simulation and experimental results show that EFD jet 3D printing achieves CISA without any prior requirements for both conductive and insulating substrate while there is no repulsion effect of the jet. The 3D highly ordered microstructures can be fabricated with the largest ratio of fiber diameter/fiber spacing of 0.29, which is much larger than other reported results, by maintaining a vertical jet and overcoming the jet deflection using a symmetrical printing path. Finally, the application of these printed 3D scaffolds with a fiber diameter of about 20 µm and a fiber spacing of 80 µm in cardiac tissue engineering has been investigated, and hiPSC‐CMs were cultured into the scaffolds to develop oriented ECTs. Immunocytochemical staining of *α*‐actinin and cTNT demonstrated that 3D‐printed scaffolds promoted the striation pattern of ECTs with one long axis along which myofibrils are regularly arranged. Moreover, ECTs developed in 3D‐printed scaffold exhibited synchronous function that was characterized by calcium imaging with synchronous transit, orchestrated frequency, and similar amplitude. Parameters to demonstrate the high efficiency of calcium handling in ECTs were also analyzed. The comparisons showed that 3D‐printed highly ordered microstructure with such fiber diameter and small fiber spacing promoted the maturation of ECTs. Therefore, the 3D printing process facilitates the fabrication of scaffolds providing an ideal microenvironment to promote the maturation of ECTs, thereby, showing great potential for application in cardiac tissue engineering.

## 3 Experimental Section

### Materials

The printing materials of PLA (PLA‐4032D powder) with an average molecular weight of 80 000 g mol^−1^ were purchased from Nature Work Co., USA.

### 3.1 Printer and Processing Parameters

A homemade EFD jet microscale 3D printer with a moving stage resolution of 1 µm was applied for printing. All printing was performed at 23 °C ambient temperature and a heating temperature of 180 °C. The nozzle to substrate distance was 250 µm, and the nozzle had an outer diameter of 200 µm with a wall thickness of 20 µm.

### Experiments for Investigating Jet Behavior and Printing Typical Microstructures

For the investigation of jet behavior, the applied voltage was set as 2 kV, air pressure of 1.8 kPa, and a printing speed of 100 mm min^−1^. The multi‐layers (10 layers, 5 layers, and 1 layer) structures were first printed on the glass and copper substrates, respectively. After printing the multi‐layers, the applied voltage was turned off and the printhead was moved to the left side with a set offset (0, 50, 100, 150, and 200 µm). afterward, the applied voltage was turned on again and the jet behaviors in the printing process were recorded by the charge‐coupled device (CCD) camera (iSpeed‐221, IX Cameras, UK). Electrostatic interaction between two fibers has been proposed based on designed fiber spacing (S*_f set_*), measured fiber spacing (S*_f measured_*), and a plot of the degree of offset metric (D*o*), which is defined as

(2) $$D_{0}=\frac{S_{fmeasured}-S_{fset}}{S_{fset}}$$

The surface potentials of printed structures with a diameter of 1 mm and a height of 500 µm on both copper and glass substrates were tested using a surface potential meter (Trek 341B). For typical microstructure printing, the processing parameters for multilayer stacked PLA structures were set as the voltage of 1.9 kV, air pressure of 2.5 kPa, a printing speed of 300 mm min^−1^, and a printhead was moved up with 6 µm after each layer. The 3D highly ordered microstructures with three different fiber spacing (100, 80, 70, and 60 µm) were printed using a symmetrical path. The morphology and microscale properties of the printed structures were characterized by field‐emission SEM (MERLIN Compact, Zeiss, Germany).

### Simulation

The COMSOL MULTIPHYSICS software was used to simulate the intensity and distribution of the electric field. Three different parameters were used: different heights of printed structure ranging from 0 to 3000 µm, different offsets with the printed structure (width and height of 20 and 60 µm, respectively), and different spacings between two symmetrical printed structures (width and height of 20 and 60 µm, respectively) on both copper and glass substrates. The simulation used the same parameters as the experimental parameters, namely, a nozzle‐to‐substrate distance of 250 µm, an outer diameter of 200 µm, a wall thickness of 20 µm, and an applied voltage of 2 kV. The medium between the nozzle and the substrate was set as air with a relative permittivity of 1, and the relative permittivity values of the copper and glass substrates were set to 1 and 4.4, respectively. The printing material was PLA with a dielectric constant of 2.5. The width and height of 20 and 60 µm, respectively.

### Differentiation of Human Induced Pluripotent Stem Cells into Cardiomyocytes

Human induced pluripotent stem cells (hiPSCs) were cultured in a chemically defined E8 medium (#1014500, Cellapy, China) on Matrigel (1:300, #356231, Corning, Tewksbury MA, USA). hiPSCs underwent differentiation towards cardiomyocytes when they reach ≈90% confluence. From the start of differentiation (D0), the E8 media is switched to cardiac differentiation media CDM3 comprised RPMI1640 (#11875500, Gibco, USA), bovine serum albumin(BSA, #9048‐46‐8, Sigma, USA), and L‐ascorbic acid (LAA, # 1713265‐25‐8, Sigma, USA). During the first two days, the cells were treated with the Gsk3*β* inhibitor CHIR99021(#252917‐06‐9, Sigma, USA) to potentiate the Wnt signaling pathway. After 48 h (D2), the cells were treated with a Wnt inhibitor C59 (#S7037, Selleck, USA) for consecutive two days. Cell beating could be seen around D8‐D9. Cardiomyocytes underwent selection and purification metabolically using CDM‐L medium comprised of glucose‐free RPMI‐1640 (#11879‐020, Gibco. USA), BSA, LAA, and L‐lactic acid (#L4263, Sigma, USA). After purification, cardiomyocytes were maintained in CDM3 medium and seeded onto a glass coverslip or into the PLA scaffold, which were pre‐coated with Matrigel at D18‐20. RPMI 1640 medium supplied with 10% FBS was used for the first 24 h culture and then the medium was changed to CDM3 for the consecutive culture. The medium was changed every other day.

### Morphological and Immunocytochemical Analysis of hiPSC‐CMs Cultured in PLA Scaffolds

The hiPSC‐CMs cultured on glass coverslip or in PLA scaffolds were harvested at 10 DIV and then fixed in 4% paraformaldehyde. The cells were then incubated with 0.1 M glycine, permeabilized with Triton X‐100 (0.1%), incubated with BSA (0.5%) in Phosphate buffer saline (PBS), and then incubated overnight at 4°C with primary antibodies: anti‐cTNT (1:300, Rabbit polyclonal to Cardiac Troponin T, ab45932), anti‐*α*‐actinin (1:300, Mouse monoclonal [EA‐53] to Sarcomeric Alpha Actinin, ab9465). Afterward, the cells were washed with PBS and the secondary antibodies (1:400, IgG Goat‐ anti‐Mouse 594 and IgG Goat anti‐Rabbit 488, A‐11005 and A‐11008, Invitrogen, USA) were incubated for 30 min. Nuclei were stained with DAPI (1:500, D9542, Sigma‐Aldrich). Finally, Vectashield (Vector Laboratories) was used to mount the samples. The staining was observed with a Zeiss confocal microscope. Analysis of the image stacks was accomplished using Fiji (http://fiji.sc/Fiji).

### Calcium Imaging

The calcium imaging acquisition procedures and analysis were reported in the previous work.^[^ ^59^, ^63^, ^64^ ^]^ In brief, hiPSC‐CMs cultures at 8 DIV were incubated with a membrane‐permeable calcium dye Fluo4‐AM (Life Technologies) in the medium for 30–45 min. Then, the medium was removed and the cultures were washed with Ringer's solution (3 mM KCl, 145 mM NaCl, 1 mM MgCl2, 1.5 mM CaCl2, 10 mM Hepes, and 10 mM glucose, pH 7.4) three times. The calcium imaging acquisitions were performed at an Olympus microscope at room temperature, and images were acquired at 20 Hz for 2 min.

### Calcium Imaging Analysis

The initial image sequences from the calcium imaging were processed with the ImageJ software, and the region of interest (ROI) was selected to acquire the time course of the fluorescence intensity, If(*t*). The calcium transients of each ROI were extracted in a semi‐automatic manner by selecting a threshold for the smallest detectable peak that was equal to three times the standard deviation of the baseline. Subsequently, the decay of If(*t*) was fitted to a cubic spline (Y(*t*)) interpolating If(*t*) at 10 or 20 points. Y(*t*) was then added to the original optical signal to compensate for dye bleaching, and the fractional optical signal was calculated as follows: DF/F = (Y(*t*) + If(*t*))/If(0), where If(0) is the fluorescence intensity at the beginning of the recording. Moreover, Tau, FWHM, Contract 90, Contract 50, Relax 50, and Relax 90 were calculated for every transient.^[^ ^58^ ^]^ The definition of the parameters was shown in Figure S8, Supporting Information.

## Conflict of Interest

The authors declare no conflict of interest.

## Supporting information

Supporting Information

Supplemental Video 1

Supplemental Video 2

Supplemental Video 3

Supplemental Video 4

Supplemental Video 5

## Data Availability

The data that support the findings of this study are available from the corresponding author upon reasonable request.
