# Supplementary material for: Electric‐Field‐Driven Printed 3D Highly Ordered Microstructure with Cell Feature Size Promotes the Maturation of Engineered Cardiac Tissues
Source: Adv Sci (Weinh). 2023 Feb 13;10(11):2206264. doi: 10.1002/advs.202206264 (PMC10104649; doi:10.1002/advs.202206264)
Supplement: Supplementary file 1 — Supporting Information [file ADVS-10-2206264-s004.pdf]

ADVANCED SCIENCE
Open Access

Supporting Information

for *Adv. Sci.*, DOI 10.1002/advs.202206264

Electric-Field-Driven Printed 3D Highly Ordered Microstructure with Cell Feature Size Promotes the Maturation of Engineered Cardiac Tissues

Guangming Zhang, Wenhai Li, Miao Yu, Hui Huang, Yaning Wang, Zhifeng Han, Kai Shi, Lingxuan Ma, Zhihao Yu, Xiaoyang Zhu, Zilong Peng, Yue Xu, Xiaoyun Li, Shijun Hu, Jiankang He, Dichen Li, Yongming Xi, Hongbo Lan, Lin Xu*, Mingliang Tang* and Miao Xiao**

Supporting information

Electric-field-driven printed 3D highly ordered microstructure with cell feature size promotes the maturation of engineered cardiac tissues

Guangming Zhang, Wenhai Li, Miao Yu, Hui Huang, Yaning Wang, Zhifeng Han, Kai shi, Lingxuan Ma, Zhihao Yu, Xiaoyang Zhu, Zilong Peng, Yue Xu, Xiaoyun Li, Shijun Hu, Jiankang He, Dichen Li, Yongming Xi, Hongbo Lan*, Lin Xu*, Mingliang Tang *, Miao Xiao*

EFD jet microscale 3D printing is very different from EHD jet printing in the aspect of action mechanisms and printing behavior. As shown in Figure S1, although only the ground electrode (a conducting substrate or mounted under an insulating substrate) in the EHD jet printing is canceled EFD jet microscale 3D printing, it is a very significant improvement. This improvement changed the generation method of the electric field for printing. The electric field in EHD is generated between two counter electrodes (nozzle electrode and ground electrode), which is like a capacitor; While the electric field in EFD is generated by the action of electrostatic induction/polarization between the nozzle tip and the top surface of the substrate. It can be seen from the distribution of electric field intensity (Figure S2). In EFD jet 3D printing method, the electric field is mainly focused on the top of the printed structure (Figure S2 a). While in the EHD jet printing method, the electric field is still affected by the countered grounded electrode when there is a printed high wall structure (Figure S2 b). In order to present the differences between electric-field-driven jet 3D printing and conventional EHD jet printing. We conducted a set of controlled experiments using the parameters (Table S1). With the stacking up of multi-layer, the electric field decreases due to the shielding in the conventional EHD jet printing, finally becoming too low to keep the jet (Figure S3 f). However, compared with conventional EHD jet printing, the jet can be kept for printing in our method (Figure S3 c).

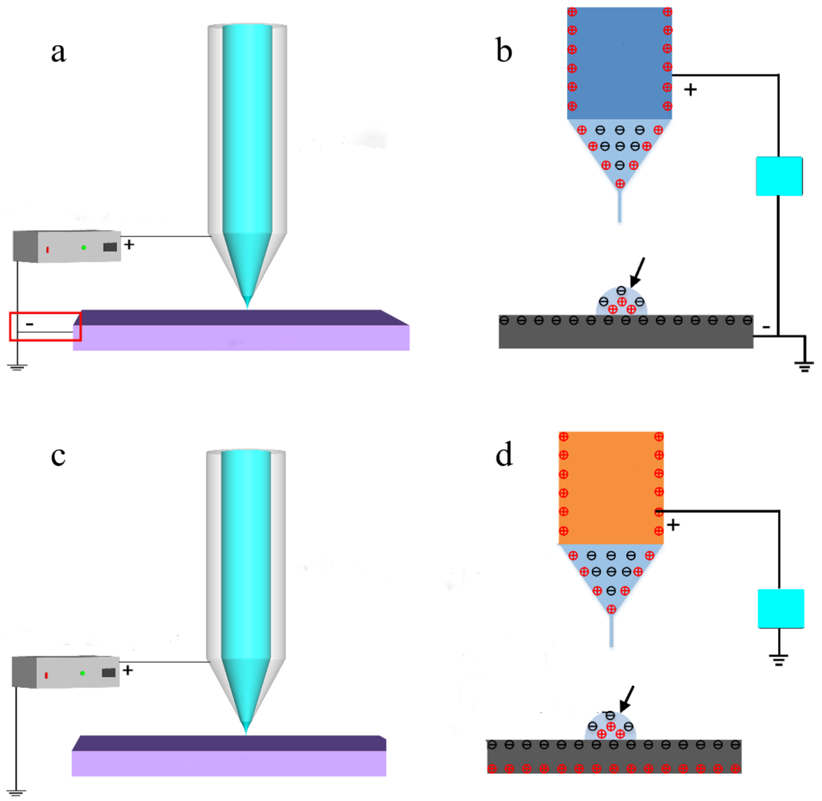

Figure S1 The comparison of EHD and EFD: a) the set-up schematic of EHD jet 3D printing; b) The charges distribution of EHD jet 3D printing; c) the set-up schematic of EFD jet 3D printing; d) The charges distribution of EFD jet 3D printing

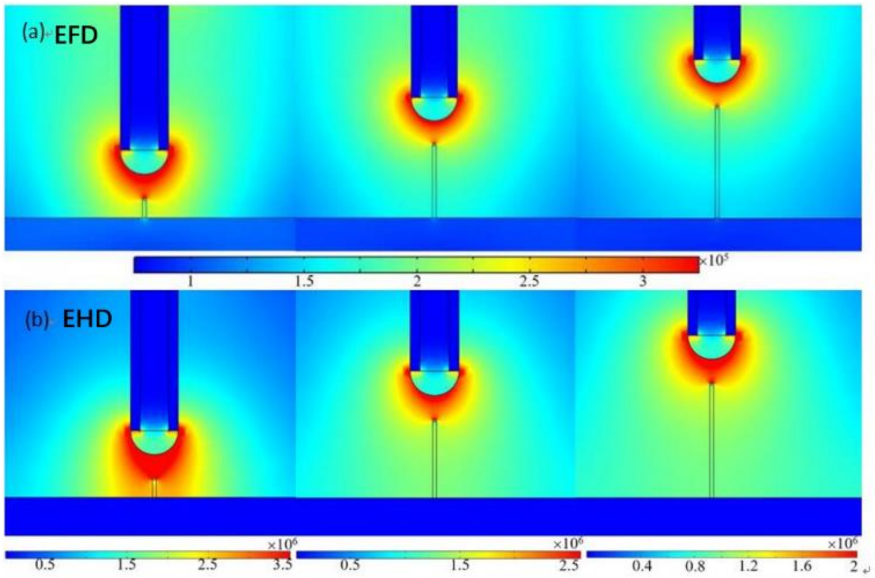

Figure S2 The distribution of electric field intensity when printing high AR wall structure with the height from 1 mm to 3 mm by: a-c) electric-field-driven jet 3D printing; d-e) conventional EHD jet printing.

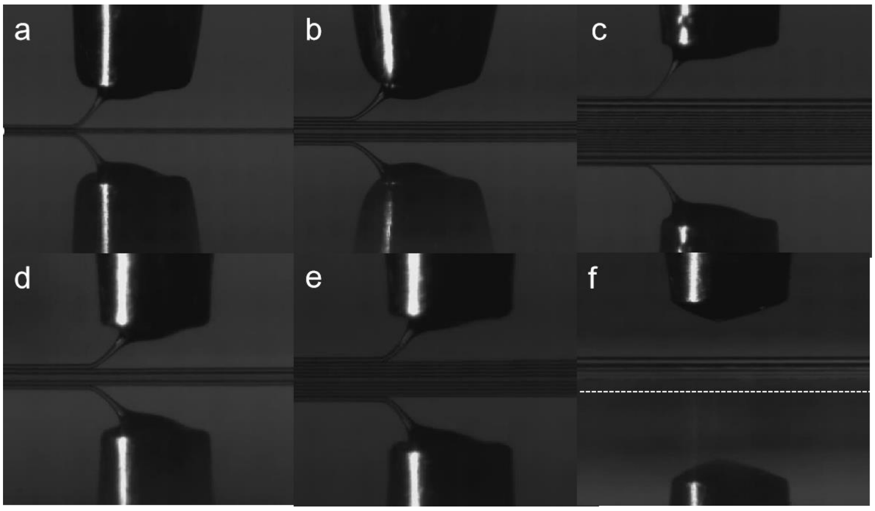

Figure S3 The high AR wall structure is printed by: a-c) electric-field-driven jet 3D printing; d-e) conventional EHD jet printing.

Table S1 The parameters of these two methods in controlled experiments

Method	Voltage [V]	Air pressure [kPa]	Printing speed [mm/s]	Standoff height [μm]	Nozzle inner diameter [μm]
Our method	1350	2.2	1	200	250
Conventional EHD	1150	2.2	1	200	250

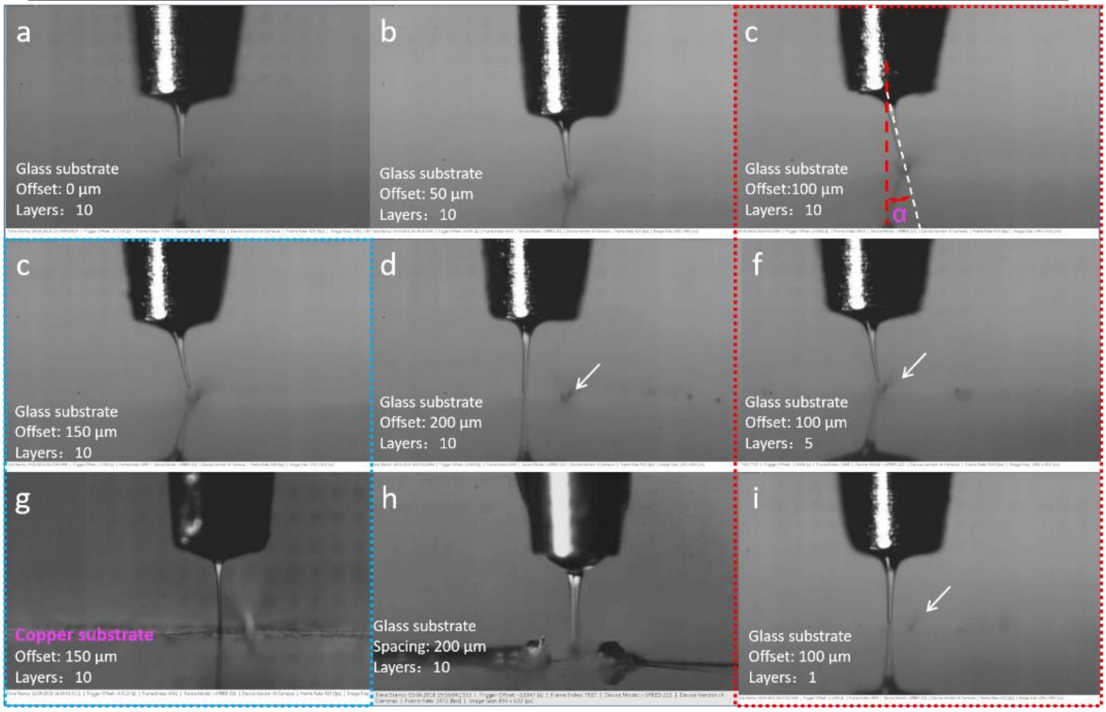

Figure S4 The jet behaviors observed by the CCD camera.

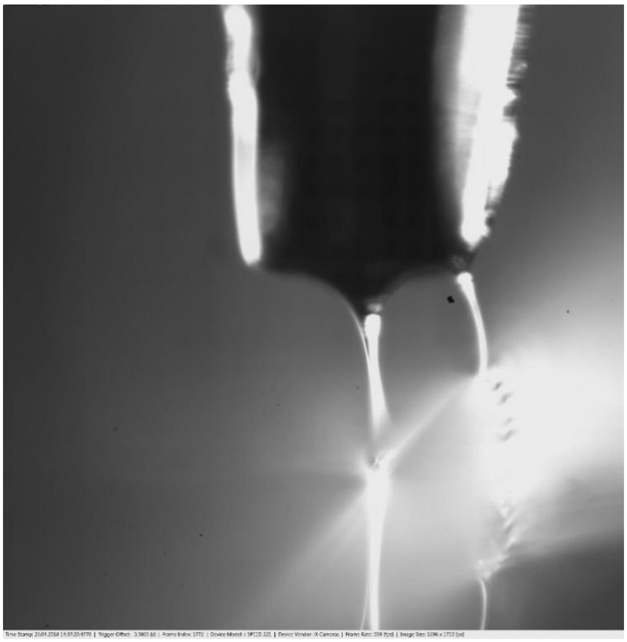

Figure S5 The multi-jet on two printed structures by the attraction force between the conductive nozzle and the top surface of the printing structure.

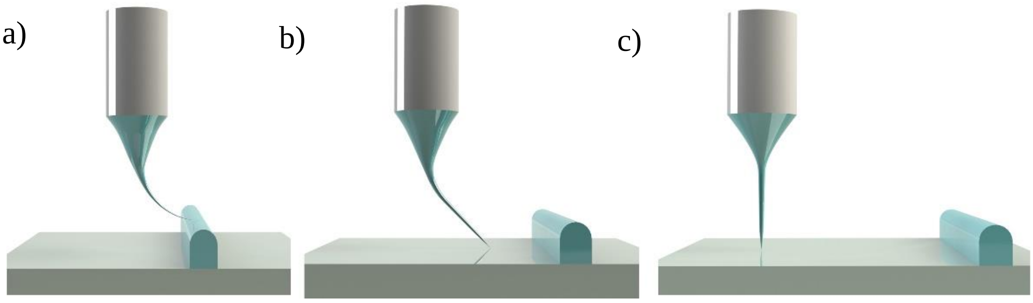

Figure S6 The three stages of the jet are dependent on the designed fiber spacing: a) the multilayer stacking by CISA, b) the jet deflection, and c) the precise deposition.

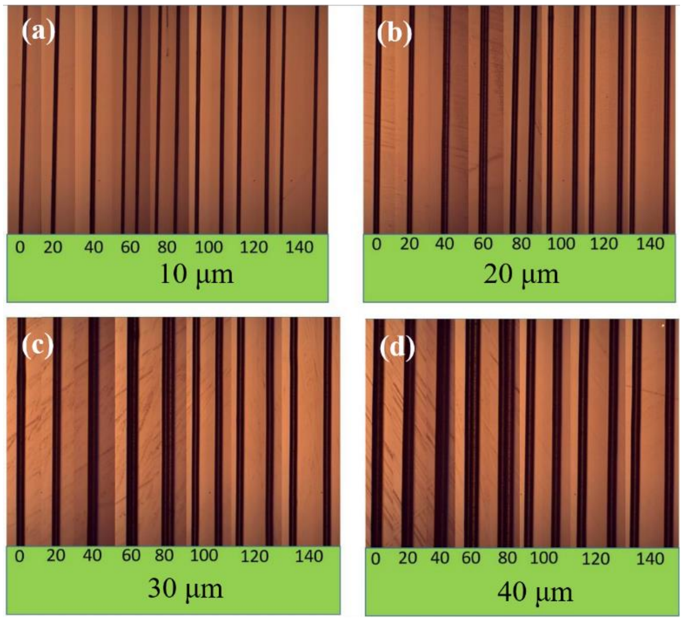

Figure S7 The effect of designed fiber spacing on the printed results of different fiber sizes. a) 10μm, b) 20μm, c) 30μm, d) 40μm.

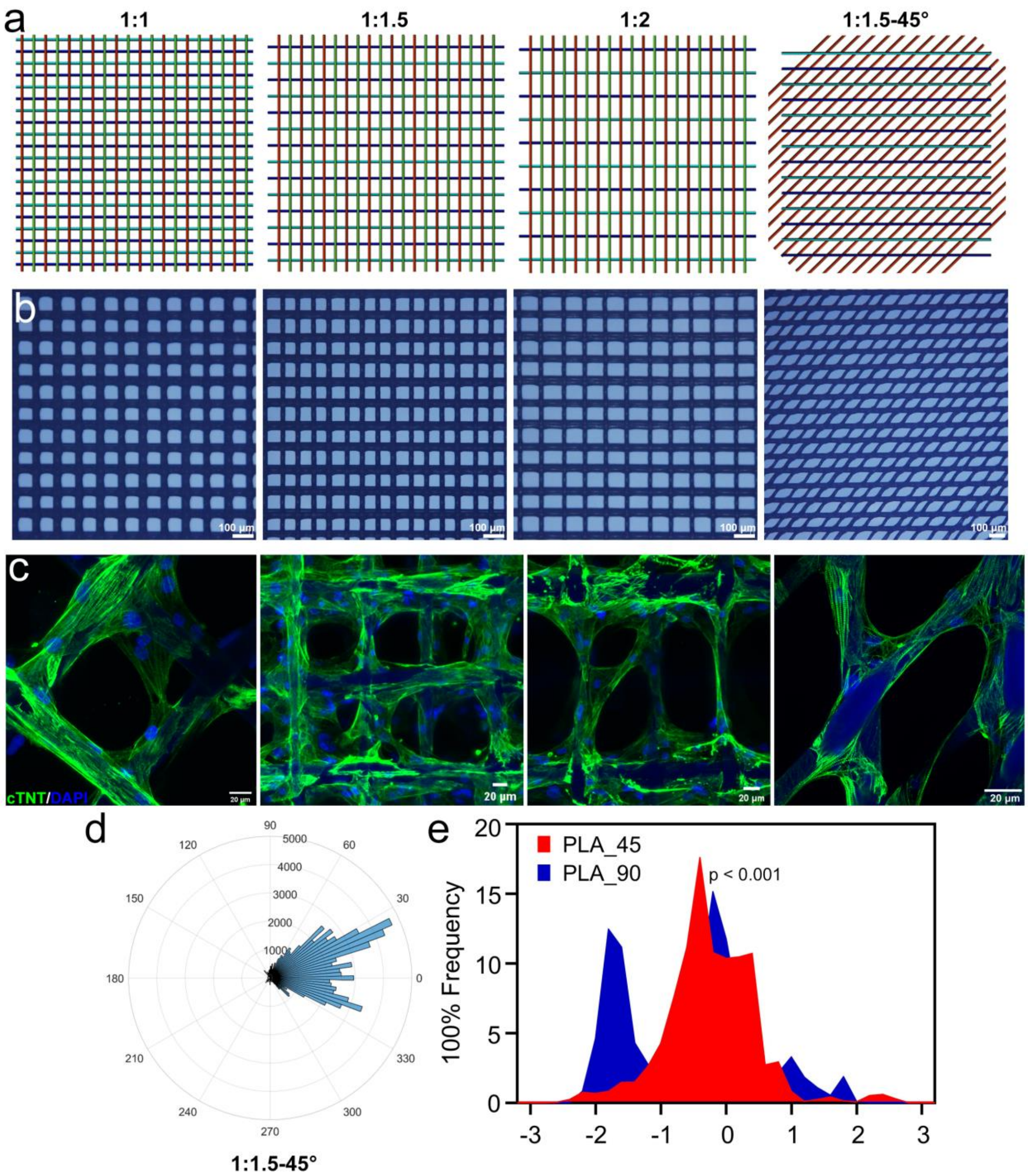

Figure S8. Regulation of the length-width ratios and angles between the layers of the 3D porous PLA scaffolds. a) Schematic diagram of scaffolds with length-width ratios of 1:1, 1:1.5, and 1:2 as well as 45° between the PLA layers. b) Scaffolds with length-width ratios of 1:1, 1:1.5, and 1:2 were fabricated. Scaffolds with 45° between the PLA layers were also fabricated. c) The hiPSC-CMs were cultured in 3D porous PLA scaffolds with different structures for 10 days. The hiPSC-CMs were stained with cardiac troponin T (cTNT), and 4',6-diamidino-2-phenylindole was used to stain the nucleus. d and e) Representative polar histogram of the sarcomeric orientation and comparison between the sarcomeric orientation distribution of the hiPSC-CMs cultured in the 3D porous PLA scaffolds with 45° and 90° (1:1.5) between the PLA layers. $p < 0.001$ from the Kolmogorov-Smirnov test.

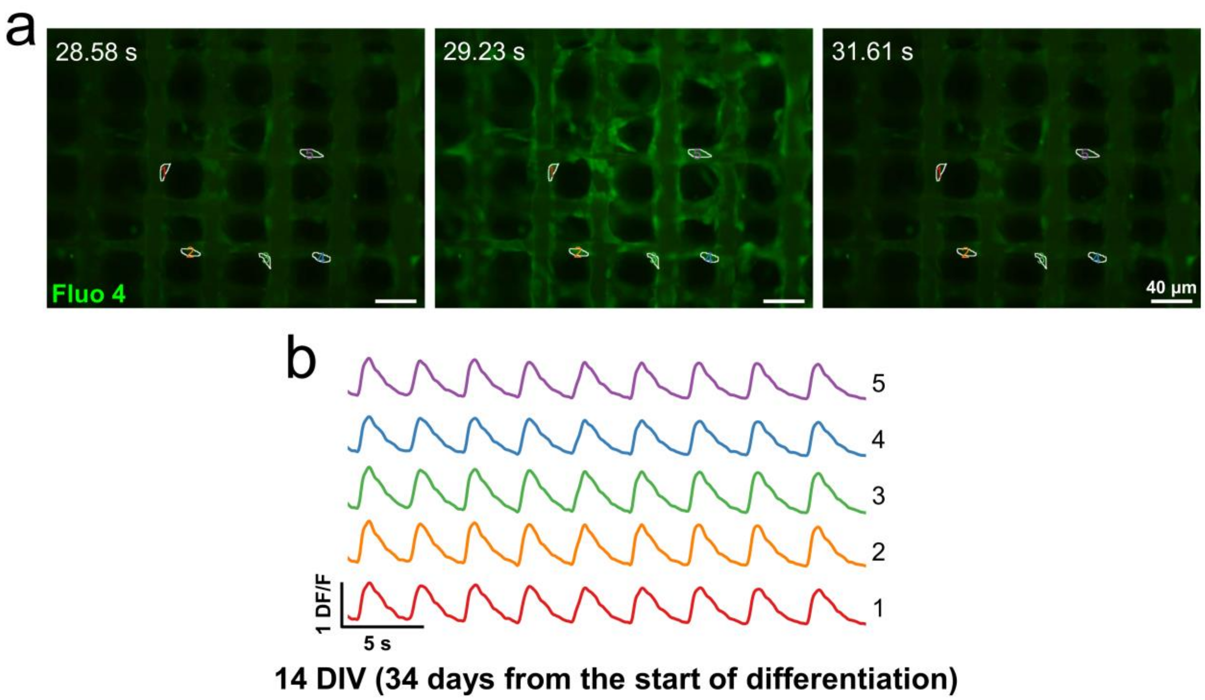

Figure S9. The ECTs showed functional synchronization when hiPSC-CMs were cultured in the scaffolds for 14 days (34 days from the start of differentiation). a) Immunofluorescence images of the cardiomyocytes cultured in the scaffolds for 14 DIV were loaded with the calcium fluorescent dye Fluo-4 AM and the synchronous calcium transients were acquired from the labeled cardiomyocytes (b).

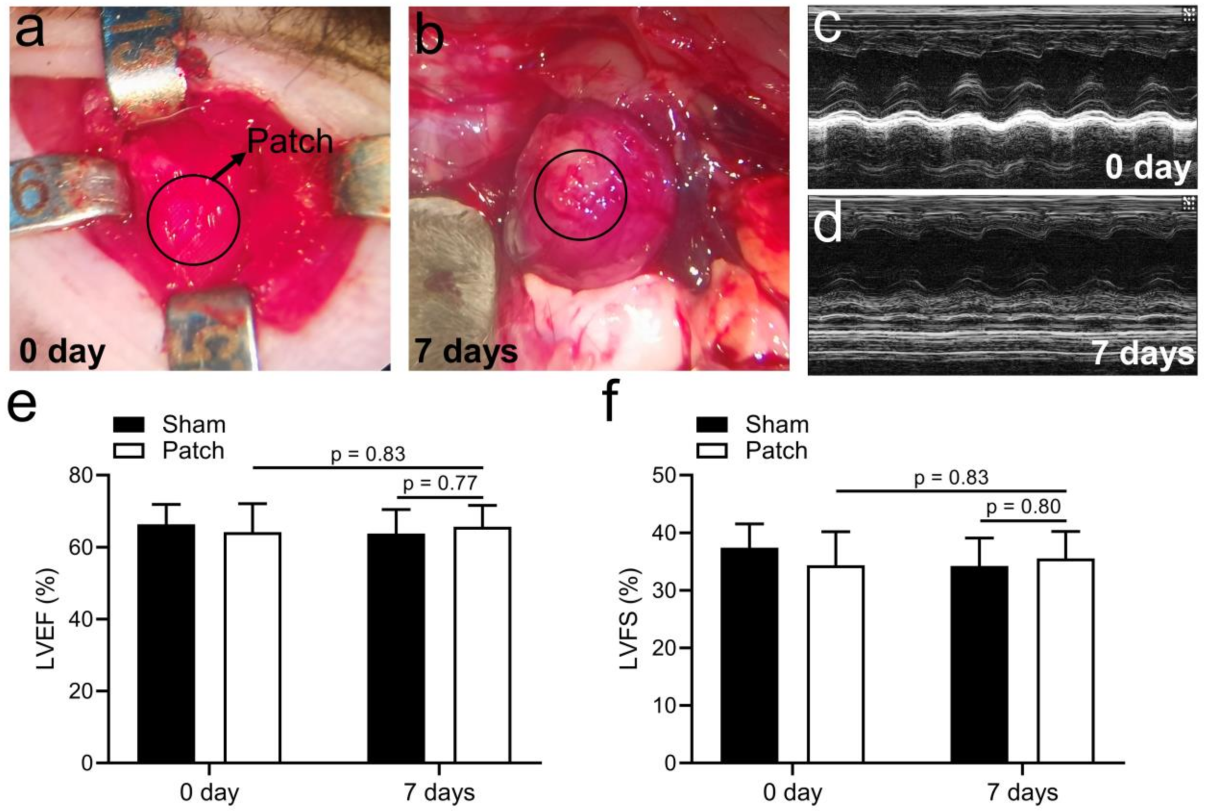

Figure S10. 3D porous PLA scaffolds were safe to animals when used as cardiac patches. The scaffolds were transplanted to the left ventricular anterior walls of mice (a). After 7 days post-transplantation, the scaffolds remained in the left ventricular anterior wall (b). The cardiac function was evaluated using echocardiography before and 7 days after transplantation (c and d). e and f) Left ventricular ejection fraction (LVEF) and left ventricular shortening fraction (LVFS) were compared.

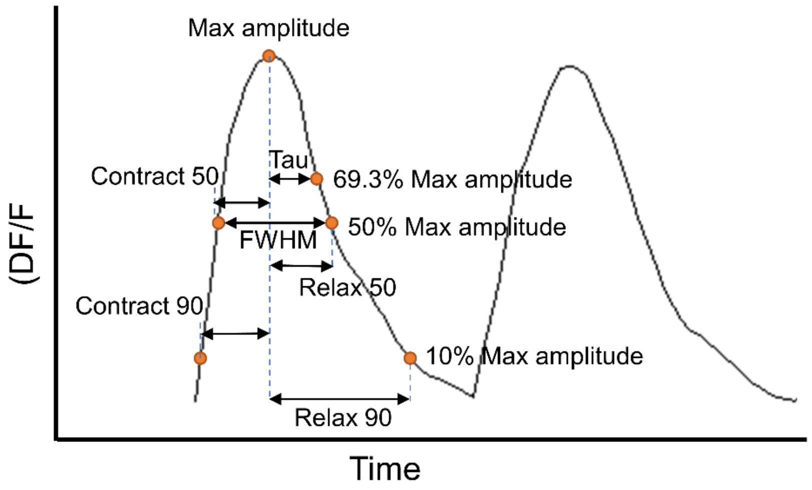

Figure S11 Parameters in the analysis of calcium transient. The parameters of FWHM (the full width half max), constant Tau during the decay, Contract50 (half time during the raise), Contract90 (90% time during the raise), Relax50 (half time during the decay) and Relax90 (90% time during the decay)

Table S2 The ratio of fiber diameter/fiber spacing with other state-of-the-art results in the literature

**Printing method**	**Materials**	**Fiber diameter (μm)**	**Fiber spacing (μm)**	**Ratio (fiber diameter/ fiber spacing)**	**Reference**
direct-writing E-jet printing	Polyvinyl Pyrrolidone(PVP) /Glycerol	0.068	1	0.068	[39]
MEW	Poly (ε-capro-lactone)-poly (ethylene glycol) (PECL)	5	50	0.1	[40]
MEW	Polycaprolactone(PCL)	5.58	100	0.0558	[41]
MEW	Polycaprolactone(PCL)	7	100	0.07	[42]
MEW	Polycaprolactone(PCL)	10	125	0.08	[43]
MEW	Polycaprolactone(PCL)	10.4	300	0.0347	[44]
EHD jet 3D printing	Polycaprolactone(PCL)/ Polyvinyl Pyrrolidone(PVP)	11	200	0.055	[45]
MEW	Polycaprolactone(PCL)	11.18	125	0.08944	[46]
EHD printing	Polycaprolactone(PCL)	11.2	100	0.112	[47]
MEW	Polycaprolactone(PCL)	12	100	0.12	[48]
MEW	Polycaprolactone(PCL)	13	200	0.065	[49]
Melt EHD printing	Polycaprolactone(PCL)	16	100	0.16	[50]
MEW	Polycaprolactone(PCL)	16.7	200	0.0835	[51]
MEW	Polycaprolactone(PCL)	18	100	0.18	[52]
MEW	Polycaprolactone(PCL)	18.45	400	0.0461	[53]
MEW	Polycaprolactone(PCL)	20	225	0.0889	[54]
MEW	Polycaprolactone(PCL)	22.9	100	0.229	[55]
MEW	Poly(lactide-block-ethylene glycol-block-lactide) (PLA-PEG-PLA)	30	500	0.06	[56]
EFD	Polylactic acid (PLA)	10	40	0.25	Our work
EFD	Polylactic acid (PLA)	20	70	0.29	Our work

Supplementary video S1. The jet deflection on the top surface of stacked 10 layers with the offset from 150 μm.

Supplementary video S2. The jet deflection restrained in the middle of a pair of symmetrical walls.

Supplementary video S3. The hiPSC-CMs cultured in the 3D printed scaffold at 8 DIV were incubated with a membrane-permeable calcium dye Fluo4-AM and the calcium transients were recorded for 1.5 min.

Supplementary video S4. Color saturation mode of the calcium imaging in Supplementary video S4.

Supplementary video S5. The hiPSC-CMs cultured in the 3D printed scaffold at 14 DIV (34 days from the start of differentiation) were incubated with a membrane-permeable calcium dye Fluo4-AM and the calcium transients were recorded for 1 min.
